# One- and Two-Photon Excited Autofluorescence of Lysozyme Amyloids

**DOI:** 10.1021/acs.jpclett.2c00570

**Published:** 2022-05-23

**Authors:** Manuela Grelich-Mucha, Maciej Lipok, Mirosława Różycka, Marek Samoć, Joanna Olesiak-Bańska

**Affiliations:** † Advanced Materials Engineering and Modelling Group, Wroclaw University of Science and Technology, Wybrzeże Wyspiańskiego 27, 50-370 Wrocław, Poland; ‡ Department of Biochemistry, Molecular Biology and Biotechnology, Faculty of Chemistry, Wroclaw University of Science and Technology, Wybrzeże Wyspiańskiego 27, 50-370 Wrocław, Poland

## Abstract

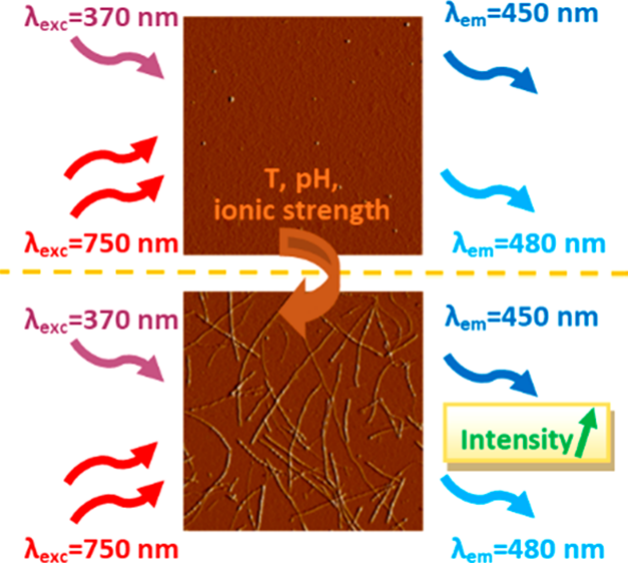

Autofluorescence properties of amyloid fibrils are of much interest but, to date, the attention has been given mostly to one-photon excited fluorescence (1PEF), while the two-photon excited fluorescence (2PEF) properties of amyloids are much less explored. We investigate 1PEF and 2PEF of hen egg-white lysozyme (HEWL) in the form of monomers and fibrils. HEWL monomers feature some autofluorescence, which is enhanced in the case of fibrils. Moreover, by varying NaCl content, we introduce changes to fibrils morphology and show how the increase of the salt concentration is linked with an increase of 1PEF and 2PEF intensities. Interestingly, we observe 2PEF emission red-shifted in comparison to 1PEF. We confirm the presence of different relaxation pathways upon one- or two-photon excitation by different lifetimes of the fluorescence decays. Finally, we correlate the changes in optical properties of HEWL fibrils and monomers with salt-mediated changes in their morphology and the secondary structure.

The name amyloid refers to peptide or protein aggregates associated with certain neurodegenerative disorders including Parkinson’s disease, Alzheimer’s disease (AD), and type II diabetes.^1−4^ Their formation can be accomplished under laboratory conditions at low pH and elevated temperatures.^2,5^ One of their peculiar properties is the presence of a common backbone architecture, i.e., secondary β-sheet structure stabilized by a hydrogen-bonding (H-bonding) network.^3,6,7^ Another interesting feature of amyloid structures is their intrinsic fluorescence.^8−16^ Upon excitation at 350–380 nm, emission occurs in the visible range of spectrum, ca. 430–450 nm.^9,13,14^ The exact mechanism of the phenomenon still remains elusive, with several hypotheses having been put forward. Some of the reported papers claim the important role of proton transfer.^8,9^ The process may include intermolecular proton transfer through H-bonds between N- and C-termini of opposite β-strands.^8^ Joseph et al. suggested not only intermolecular but also intramolecular proton transfer along H-bonds in the excited state.^9^ Intrinsic fluorescence may also result from inter- and intrachain charge-transfer excitations through H-bonds.^10^ Recently, it has been reported that the autofluorescence is significantly affected by specific H-bonding network and especially enhanced by short H-bonds.^12^ Grisanti et al.^11^ reported on the role of the multitude of nπ* states and efficient hindering of nonradiative relaxation pathways by the β-sheet structure. Another mechanism explaining amyloid autofluorescence may be aggregation-induced emission (AIE). Progressive aggregation may lead to increase of inter- and intramolecular interactions and thus to increased fluorescence intensity.^17−20^ Overall, in the literature many hypotheses explaining the possible mechanism responsible for autofluorescence properties of amyloid structures have been formulated. The role played by H-bonding network stabilizing the β-sheet structure is especially emphasized. As we mentioned above, the H-bonding network along with short hydrogen bonds^12^ is conducive to proton or charge transfer.^8−10^ Short hydrogen bonds stiffen the H-bonding network which as a result promotes radiative relaxation.^12^ The H-bonding network supports the nπ* states which possibly also affect the autofluorescence properties.^11^ There are several reports claiming that protein monomers also possess autofluorescence properties. However, monomer-to-fibril aggregation induces enhancement of the optical properties. Niyangoda et al.^21^ observed enhancement in fluorescence intensity of HEWL fibrils compared to their monomeric counterparts. They assumed that carbonyl groups were the source of the observed fluorescence. However, not only the carbonyl groups may be responsible for intrinsic fluorescence properties. Again, the multiple hydrogen bonds N–H···O=C present in the structures and also short contacts including H–C···O=C, N–H···C=O, N–H···C–H, H–N···N–H, or H–N···O=C which stabilize the structure of HEWL and other proteins can play a crucial role.^17^ Concentration- and aggregation-induced emission was noticed for bovine serum albumin (583 amino acids (AAs), MW ∼ 66 kDa)^17^ and human serum albumin (585 AAs, MW ∼ 66.5 kDa)^22^ solutions. Bhattacharya and co-workers^22^ reported that intrinsic fluorescence can arise from oligomeric and not from monomeric human serum albumin.

With the need of the detection and imaging of amyloids in tissues and organisms, employing autofluorescence excited in the near-infrared (NIR) rather than by the ultraviolet (UV) may be beneficial. Wavelengths from the NIR region, i.e., biological window, penetrate tissues deeper compared to the wavelengths from the UV region.^23−26^ Because excitation in the NIR must involve multiphoton absorption, it is desirable to explore the nonlinear optical properties of amyloid structures. Kwan et al.^27^ reported on intrinsic fluorescence from brain slices of AD transgenic mouse using multiphoton and second-harmonic generation (SHG) microscopies. Johansson and Koelsch,^28^ using the same techniques, detected intrinsic fluorescence from spherical amyloid structures, i.e., spherulites. Strong multiphoton absorption, which depends on the wavelength of light, was also evidenced for amyloid structures of various proteins.^29^ Recently, our team has reported that two-photon excited autofluorescence of amyloid spherulites is polarization-dependent and may be a promising tool in determination of the organization of fibrils.^14^ These results evidence sizable nonlinear optical properties of amyloid structures and hold promise for detecting them without the need for any extrinsic two-photon excited fluorophore.

Here, we report the one- and two-photon excited autofluorescence (1PEF and 2PEF) spectra of amyloids with various morphologies. We investigated a lysozyme mutant, hen egg-white lysozyme (HEWL), which constitutes an attractive model protein for amyloid fibrils investigations due to well-studied *in vitro* protocols leading to their formation.^30,31^ The conversion of HEWL into amyloid fibrils is favored under the conditions of elevated temperatures and low pH.^30,32−35^ We analyzed the secondary structure of HEWL monomers and fibrils obtained at various salt concentrations by using attenuated total reflectance Fourier-transform infrared (ATR-FTIR) spectroscopy. Then, we explored differences between one- and two-photon excited autofluorescence emitted by various HEWL structures. To better understand changes in autofluorescence observed for the samples, we performed one- and two-photon excited fluorescence lifetime measurements of amyloid fibrils. Our results highlight the differences between one- and two-photon excited autofluorescence of HEWL amyloids as well as the influence of fibrils morphology on their optical properties.

*Amyloid Morphology and Structure*. To induce amyloid fibrils of various morphology, but preserve the amino acid sequence, we incubated hen egg-white lysozyme (HEWL) at varying NaCl concentration (Table 1 and Figure 1). Atomic force microscopy (AFM) imaging revealed that the ionic strength has influence on the mean height and width distribution of fibrils (Figure 1, Table 1, and Figure S1): with the increase of salt concentration the mean width and height values increased by ∼0.7 nm and ∼5.6 nm, respectively. Additionally, we performed a *t* test to verify differences in mean height and width values among the samples incubated at varying salt content. Our calculations evidence that the mean heights do not differ significantly among fibrils incubated at 0 and 5 mM NaCl (Table S1), but differences between 0 and 50 mM NaCl and 5 and 50 mM NaCl are statistically significant. In the case of mean widths, differences between 0 and 5 mM NaCl and 0 and 50 mM NaCl are significant. In the case of mean widths, differences between 0 and 5 mM NaCl and 0 and 50 mM NaCl are significant, whereas between 5 and 50 mM NaCl they are not. The tests confirm that the increase of salt concentration significantly affects the morphology (widths and heights) of fibrils. Moreover, we have observed that the fibrils obtained in the absence of any salt were long and flexible, and increasing the salt concentration resulted in significantly shorter fibrils. These observations indicate the role of electrostatic interactions in the morphology of amyloid structures. The isoelectric point of HEWL is ∼10.7.^36^ In our protocol the pH of the solutions is ∼1.5. Hence, HEWL is positively charged, and between monomers, repulsive Coulombic interactions dominate. The increased concentration of salt causes screening of the electrostatic repulsion.^37,38^ With the increase of the ionic strength, fibrils tend to stick together,^38^ which is observable by comparing the fibrils incubated in the absence of any salt and at higher salt concentrations (Figure 1).

**Table 1:** Height and Width Analysis of Amyloid Fibrils Obtained at Varying NaCl Concentrations (0, 5, and 50 mM)^a^

sample	height (nm)	width (nm)
0 mM NaCl	2.0 ± 0.7	18.1 ± 5.1
5 mM NaCl	2.2 ± 0.6	22.3 ± 4.4
50 mM NaCl	2.7 ± 1.1	23.7 ± 4.0

a The average height and width values were calculated according to 50 profiles from different amyloid fibrils. To estimate the height, the highest values of measured profiles were considered. To calculate the width values, the full width at half-maximum (FWHM) method was used.

**Figure 1:**
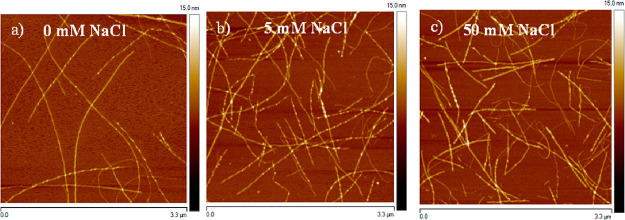
Height AFM images performed for amyloid fibrils obtained from HEWL incubated at varying salt concentration: 0 mM (a), 5 mM (b), and 50 mM NaCl (c). The size of the images is 3.3 μm × 3.3 μm, and the height contrast is set to 15 nm.

We have also investigated if increasing salt concentration influences the morphology of the studied samples before the incubation. The AFM images (Figure S2) show similar small aggregates (with the size in the range from ∼25 to ∼100 nm) in all samples, indicating that even the highest measured salt concentrations have no impact on HEWL morphology before the incubation. The theoretical molecular weight (MW_theor_) of HEWL was calculated according to the amino acid sequence given by the producer and was equal to 14.30 kDa. According to this value, sedimentation velocity analytical ultracentrifugation (SV AUC) analysis was performed. SV AUC was done for three different concentrations of HEWL, namely, 0.2, 2, and 20 mg/mL dissolved in HCl solution (pH ∼ 1.5) containing 0, 5, or 50 mM NaCl. The outcomes indicate predomination of HEWL monomers in the samples before the incubation period (Table S2 and Figure S3). Interestingly, we observed that with the increasing HEWL concentration, the sedimentation coefficient (*s*) was increasing as well (Table S2). The same tendency was reported by Wu et al.^39^ In parallel, we observed an increase of *f*/*f*_0_ (frictional ratio) values (Table S2). It means that the increased HEWL concentration leads to the change of its conformation, but still the predominant form of the protein is a monomer.

The measurements of attenuated total reflectance Fourier-transform infrared (ATR-FTIR) spectra performed for HEWL fibrils revealed some differences in the amide I band: an increase of β-sheet structure content with the increase of salt concentration, as indicated by the green arrow in Figure 2. The maximum band absorption evidencing the presence of a β-sheet structure was recorded at 1629, 1625, and 1625 cm^–1^ for the samples incubated at 0, 5, and 50 mM NaCl, respectively. The obtained results show that the β-sheet content of amyloid fibrils can be modulated by variation in the concentration of sodium chloride during incubation. Despite the secondary β-sheet structure, fibrils also possess a relatively high content of α-helical structure with maximum absorption band located at 1649, 1651, and 1649 cm^–1^, for fibrils obtained after incubation in 0, 5, and 50 mM NaCl (Figure 2).^40^ Analysis of ATR-FTIR spectra recorded for HEWL before the incubation showed that the initial HEWL aggregates are mainly composed of α-helix (Figure S4), which is in good agreement with data reported by Foley et al.^41^ For HEWL monomers dissolved in 0 and 5 mM NaCl the maximum absorption band is located at 1651 cm^–1^, whereas for the monomers dissolved in 50 mM NaCl the maximum is shifted to 1649 cm^–1^ (Figure S4).

**Figure 2:**
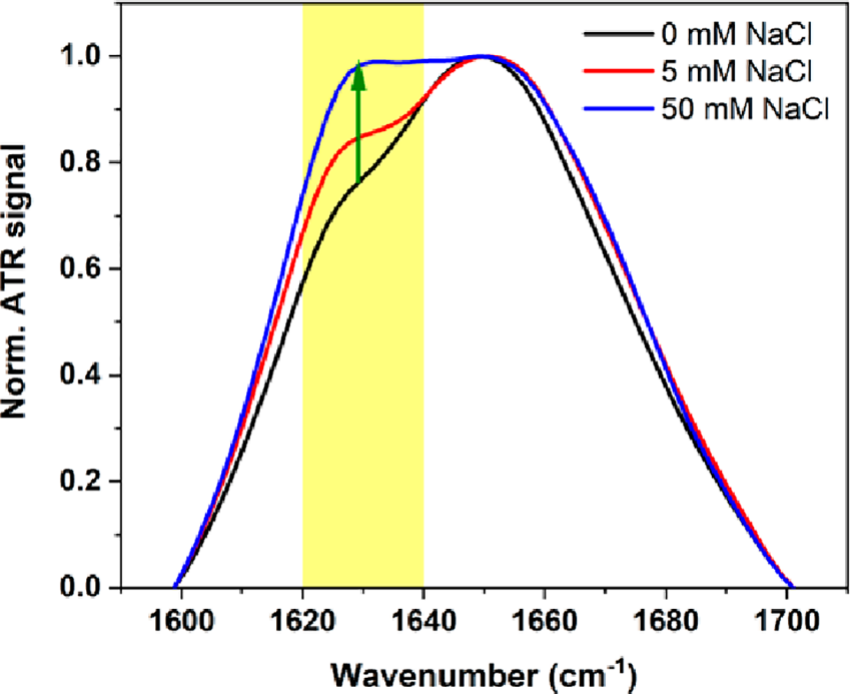
ATR-FTIR spectra recorded for HEWL fibrils incubated at varying salt concentration: 0 mM (black), 5 mM (red), and 50 mM NaCl (blue). The absorption band evidencing the presence of a β-sheet structure is indicated by a yellow rectangle (1620–1640 cm^–1^).

*One-Photon Optical Properties of Amyloids and Monomers*. We collected emission spectra (excited at 370 nm) for both HEWL monomers and target fibrils, as presented in Figure 3a. Both as-formed fibrils and preincubated samples presented fluorescence with the maximum at ∼450 nm (at 0, 5, and 50 mM NaCl the maximum emission of fibrils was localized at 451, 450, and 450 nm, respectively, while in preincubation samples the maximum was at 450 nm, independent of the salt content). Increasing NaCl concentration resulted in samples with slightly enhanced intensity of fluorescence emission. Simultaneously, we measured absorption spectra and observed the increase in a range from 320 to 410 nm (Figure S5), whereas a broad tail of absorption was extending to longer wavelengths. The recorded absorption spectra are in a good agreement with data reported by Ansari et al.^42^ Those authors assumed that the increasing intensity in absorption spectra related to the formation of HEWL aggregates is due to charge-transfer (CT) transitions. These absorption spectra are termed as protein charge-transfer spectra (ProCharTS) and result from interactions between charged residues in proteins including *inter alia* −NH_3_^+^ in Lys and −COO^–^ in Glu.^42,43^ Moreover, a significant increase in absorption-tail intensity recorded for the sample incubated at 50 mM NaCl can be explained taking into account the recorded ATR-FTIR spectra (Figure 2). This sample had the highest content of the β-sheet structure, which suggests a higher amount of amyloid fibrils, enhancing the scattering of the incoming light. To evaluate the possible contribution of light scattering in the characteristics of the emission, we measured emission spectra for the samples diluted to 2 mg/mL (10-fold dilution) (Figure S6). Normalized emission spectra for a given salt concentration overlap, independent of the sample concentration.

**Figure 3:**
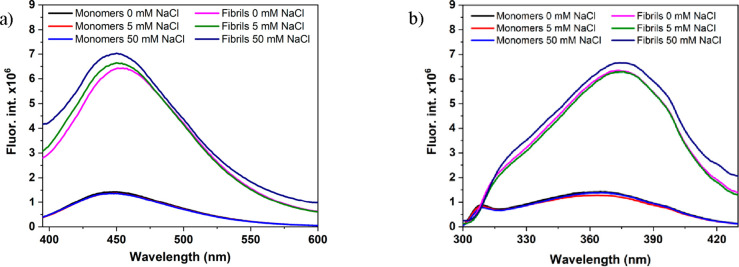
Fluorescence emission (a) and excitation (b) spectra recorded for monomers and fibrils obtained from HEWL incubated at 0, 5, and 50 mM NaCl. Emission spectra were recorded at λ_exc_ = 370 nm, whereas excitation spectra at λ_em_ = 460 nm.

Excitation spectra, recorded at λ_em_ = 460 nm for the samples after incubation at 0, 5, and 50 mM NaCl, had their maxima centered at 373, 376, and 374 nm, respectively (Figure 3b). Comparing fluorescence emission intensities between samples of HEWL before and after incubation, the results received for fibrils evidence ∼5-fold higher intensity than those from HEWL monomers (Figure 3a,b). Figure 3b shows clearly that the strength of the excitation band at λ_exc_ = 370 nm, associated with intrinsic fluorescence of fibrils, significantly exceeds that of the band assigned to aromatic amino acids (at λ_exc_ ∼ 310 nm, arising mostly from six Trp residues present in native HEWL). For comparison, in excitation spectra attributed to HEWL monomers, the excitation band assigned to aromatic amino acids is clearly visible and is of magnitude comparable with excitation band at 370 nm.

We have also measured fluorescence quantum yield (FQY) values of HEWL monomers and amyloids. Amyloids presented several times higher FQY: in the case of samples in 0 mM NaCl the FQY of monomers and amyloids was 1.23% and 4.88%, respectively. Interestingly, at 50 mM NaCl, the FQY of both monomers and amyloids increased to 2.63% and 5.31%.

*Nonlinear Optical Properties of Amyloids and Monomers*. It has been reported that amyloid structures show unexpectedly strong nonlinear optical properties.^14,28,29^ However, the information about multiphoton excited autofluorescence spectra of amyloids is scarce. We have measured two-photon excited fluorescence (2PEF) of monomers and amyloid fibrils using femtosecond laser excitation at wavelengths from 730 to 950 nm. The highest fluorescence intensity for all samples was obtained upon excitation at 750 nm (Figure 4a). The intensity of two-photon excited autofluorescence spectra collected from fibrils in 50 mM NaCl was significantly higher than that from samples incubated at lower salt concentration. Moreover, monomer samples exhibited significantly lower autofluorescence with maximum localized in the same energy region as that for fibrils, with fluorescence intensity independent of salt concentration (Figure 4a). We confirmed that the measured autofluorescence was of multiphoton origin with the dependence of 2PEF intensity on the excitation power. The values of the power exponent *n* (eq 1) obtained for femtosecond laser excitation of fibrils incubated at 0 mM NaCl and 50 mM NaCl were equal to 1.8 and 2.2, respectively, confirming the two-photon origin of the observed autofluorescence. Furthermore, the highest values of *n* factor for monomers were also seen at 750 nm and were equal to 1.7 and 1.6 for sample at 0 mM NaCl and 50 mM NaCl, also indicating the occurrence of two-photon processes. However, the differences between the sample before and after the incubation, especially the ones prepared at high salt concentration, show that fibrillation is increasing the probability of two-photon absorption. To quantify the two-photon absorption (2PA) of our samples, we determined the effective two-photon absorption cross sections (σ_2,eff_), using the fluorescence-based method, with fluorescein at alkaline conditions as a reference and employing eq 2.^44^ The values of σ_2,eff_ at 750 nm were 0.15 GM and 0.18 GM for HEWL fibrils incubated at 0 and 50 mM NaCl, respectively (Figure 4b). HEWL monomers presented significantly lower values; at 750 nm σ_2,eff_ values reached 0.08 GM for both 0 mM NaCl and 50 mM NaCl samples (Figure 4b). While these values are not very high, they are comparable with effective two-photon absorption cross section of some fluorescent dyes.^44^ Taking into account low values of FQY determined for 1PEF, the two-photon absorption cross section of HEWL amyloids is ∼3 GM (per one protein molecule), which is within the same order of magnitude as Coumarin 153, perylene, or Lucifer Yellow.^45^ However, these estimations should be taken with a grain of salt because they are based on the assumption that fluorescence quantum yields resulting from both one- and two-photon absorption are the same and the relaxation proceeds from the same energy levels. In fact, as we discuss further in the text, the two-photon excited fluorescence exhibited by lysozyme amyloids appears to result from different energy levels than the 1P autofluorescence.

**Figure 4:**
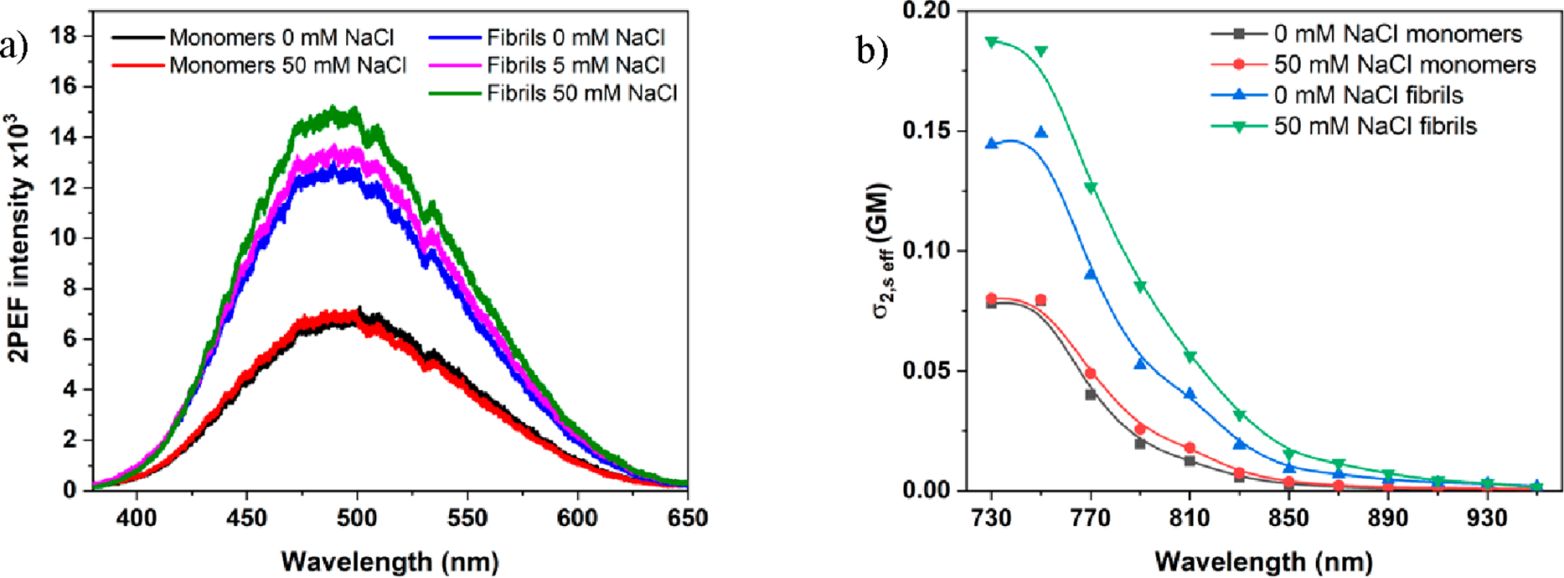
Two-photon excited emission spectra recorded for HEWL monomers at 0 mM (black) and 50 mM NaCl (red) and for HEWL fibrils at 0 mM (blue), 5 mM (magenta), and 50 mM NaCl (green) (a). Effective two-photon absorption cross section recorded for HEWL monomers incubated at 0 mM (black) and 50 mM NaCl (red) and for HEWL fibrils incubated at 0 mM (blue) and 50 mM NaCl (green).

Previous Z-scan measurements on amyloid samples provided a two-photon absorption maximum at ∼550 nm, in the range of expected two-photon absorption of aromatic amino acids,^29^ while 3PA or higher-order nonlinear optical processes were found to be dominant in the 750–950 nm range. However, the present fluorescence-based experiments and some previous reports^8,28^ have shown the presence of only two-photon processes at 750 nm. This discrepancy is likely the result of two factors: First, the occurrence of multiphoton absorption depends on the pulse width and intensity of the laser light, which may lead to inherent differences between Z-scan measurements performed with low repetition rate (1 kHz), amplified femtosecond pulses of high intensity, and 2PEF recorded with mode-locked trains (80 MHz) of femtosecond pulses, by using relatively low intensities, but at high repetition rate.^46^ Second, the Z-scan measures the variation of transmittance of the sample, which not only originates from multiphoton absorption but also may involve other processes, including for example excited state absorption or nonlinear scattering, while 2PEF records only those events that result in the emission of fluorescence. Both techniques indicate, nevertheless, that nonlinear optical properties of amyloid fibrils are strongly enhanced compared to their monomeric state.

*One-Photon vs Two-Photon Excited Fluorescence*. The striking difference between autofluorescence excited via one- and two-photons is a red-shift of 2PEF emission, as compared to 1PEF, which is clearly visible in Figure 5 and Table S3. However, the one- and two-photon excitation spectra overlap (when the one-photon spectrum is plotted at doubled wavelength) (Figure 5), which suggests that in both cases the absorption transition occurs to the same excited state. However, the differences between one- and two-photon excited emission spectra suggest fluorescence emission occurring from different electronic levels. To seek the explanation of observed red-shifted emission, we checked the factors that may induce it. We confirmed (by measurements of 1PEF and 2PEF of fluorescein) that the observed red-shift is not due to the equipment artifacts. Considering the contribution of high concentration of samples to the variations of relaxation pathways (by possible charge and energy transfer processes), we checked 2PEF of fibril samples diluted from 20 to 2 mg/mL. The 2PEF spectrum maximum for the diluted sample was located at the same wavelength (Figure S7), while, as expected, with much lower intensity. Finally, we performed one-photon (1P) and two-photon (2P) excited fluorescence decay measurements. 1P fluorescence decays were best fitted by using a triple-exponential function, while 2P fluorescence decays were best fitted with a double-exponential one. Apart from the differences in the fitting function, the results (Table 2 and Figure S8) demonstrate clearly that 1PEF has a significantly longer average lifetime (τ_av_) (eq 3) than that for 2PEF, for both samples, incubated at 0 and 50 mM NaCl. These outcomes support the existence of diverse relaxation pathways of 1PEF and 2PEF, which can cause differences observed in the steady-state spectra described in Figure 5. To verify if the observed differences in 1PEF and 2PEF are characteristic for HEWL or are general for amyloid structures, we checked the shift between 1PEF and 2PEF exhibited by bovine insulin amyloid fibrils. After collecting spectra with excitation wavelengths equal to 375 and 750 nm for 1PEF and 2PEF, respectively, we observed a significant red-shift (∼50–60 nm) (Figure S9). Thus, the red-shift between 1PEF and 2PEF of amyloids is not specific only to HEWL, but rather is a characteristic feature of fibrils of amyloidogenic proteins.

**Figure 5:**
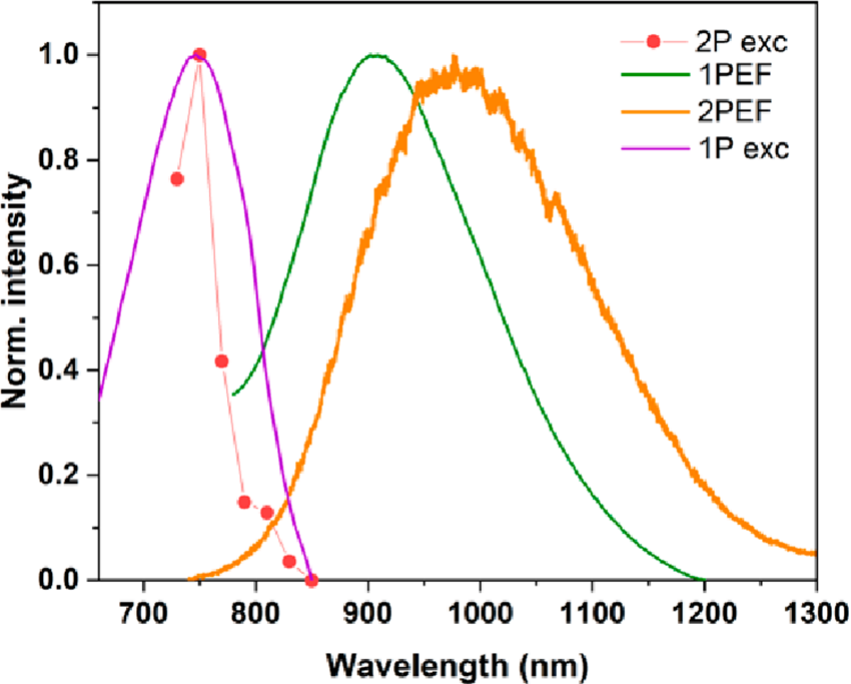
Correlation between normalized two-photon excitation spectrum (2P exc) (red circles and the dotted line) for λ_em_ = 550 nm, normalized one-photon excitation spectrum (1P exc) (violet line) for λ_em_ = 460 nm, normalized one-photon emission spectrum (1PEF) (green line) for λ_exc_ = 370 nm, and normalized two-photon emission spectrum (2PEF) for λ_exc_ = 750 nm for HEWL fibrils obtained after incubation at 0 mM NaCl. 1PEF, 2PEF, and 1P exc spectra were plotted at double wavelength compared to the 2P exc spectrum.

**Table 2:** Average Fluorescence Lifetimes (τ_av_) Calculated from One-Photon (1P) and Two-Photon Excited Fluorescence Decays (2P) of HEWL Fibrils after Incubation in 25 mM HCl at 0 and 50 mM NaCl

	τ_av_ (ns)	
NaCl concentration (mM)	1P	2P
0	2.87	1.16
50	2.99	2.08

A closer look at the behavior of one- and two-photon excited lifetimes of HEWL amyloids reveals that the observed increase of average 1PEF lifetimes with increasing sodium chloride concentration may be attributed to the increasing share of the longest lifetime calculated by using the triple-exponential function (Figure S8a,b). On the other hand, the increase of 2PEF lifetimes appears to be related to a noticeable increase in one of the calculated lifetimes from 1.3 to 2.5 ns (Figure S8c,d). This may be a result of internal changes of electronic structures of amyloids caused by a high concentration of sodium chloride.

*Autofluorescence Enhancement upon Fibril Formation*. We observed that HEWL monomers exhibit relatively weak autofluorescence. Its presence may result from CT transitions in absorption spectra.^47^ HEWL is composed of a total of 129 amino acids (AAs), among which 27 contain electrically charged side chains (∼21%).^42,47^ The fluorescence emission from HEWL monomers scales with their concentration (Figure S10). However, upon monomer-to-fibril formation these optical properties are enhanced. Moreover, increasing salt concentration also contributes to an increase in autofluorescence intensity (Figures 3 and 4b). The observed increase in intrinsic fluorescence intensity (Figures 3 and 4b) can be linked with HEWL aggregation into ordered amyloid fibrils. Upon aggregation HEWL conformation changes from α-helical in monomers (Figure S4) into fibrils characterized by the presence of a β-sheet structure (Figure 2). As-formed β-sheet structure is stabilized by mutliple H-bonds which enable the possible proton or charge transfer and stabilize the nπ* states which potentially also contribute to the amyloid’s autofluorescence.

We analyzed the fluorescence enhancement coefficient (*F*_enh,coeff_) as a function of sodium chloride concentration for HEWL fibrils and monomers (Figure 6). As the concentration of our samples was relatively high, the measured fluorescence intensity values were corrected for the possible inner filter effect, according to eq 4. Then, we calculated *F*_enh,coeff_ by dividing a given corrected fluorescence value by the corrected fluorescence value calculated for appropriate sample at 0 mM NaCl (eq 5). The outcomes demonstrate that the increase of salt concentration causes the increase of *F*_enh,coeff_ in both 1PEF and 2PEF of HEWL fibrils. The presence of 5 mM sodium chloride enhanced 1PEF_cor_ and 2PEF_cor_ by a factor of 1.3 and 1.2, while the highest salt concentration enhances it further by 3.2 and 2.9, respectively. However, for the monomers almost no increase was observed (Figure 6). We confirmed that in the case of HEWL, even at 1.4 mM (20 mg/mL), the monomeric species predominate (Table S2 and Figure S3). Thus, the observed enhancement in 1PEF and 2PEF in the case of fibrils and with the increase NaCl content suggests the critical role of β-sheet structure in the fluorescence mechanism.

**Figure 6:**
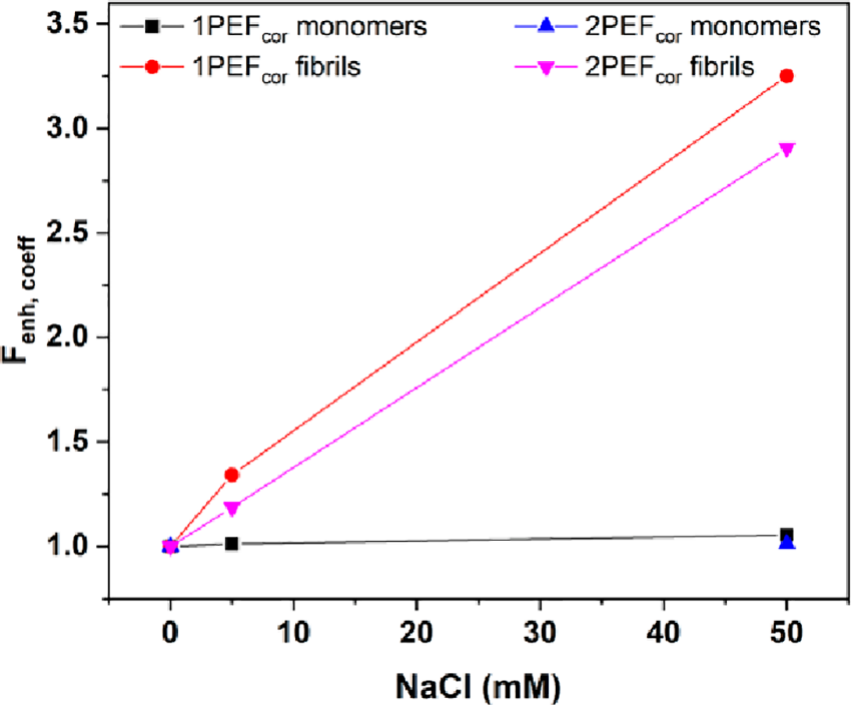
Fluorescence enhancement coefficient (*F*_enh,coeff_) of HEWL monomers and fibrils as a function of sodium chloride (NaCl) concentration for 1PEF and 2PEF processes. 1PEF_cor_ and 2PEF_cor_ correspond to corrected fluorescence values obtained upon excitation at 370 and 750 nm, respectively.

In this work one- and two-photon excited autofluorescence properties of HEWL monomers and fibrils were studied at the varying NaCl content, which provided fibrils of varying morphology and β-sheet content. Our findings indicate that both the monomers and fibrils present 1PEF and 2PEF emission in the 450–520 nm wavelength range; however, the intensity of autofluorescence is significantly lower in the case of monomers. We determined and compared the effective two-photon absorption cross sections (σ_2,eff_) of amyloid fibrils and the initial monomers, which gives a quantitative comparison between their multiphoton excitation efficiencies. We noticed that upon fibrillation the values of σ_2,eff_ increase which means that the probability of two-photon absorption is increasing as well. Moreover, calculated fluorescence quantum yields of HEWL fibrils were higher compared to their monomeric counterparts. Moreover, not only transition from monomeric species into fibrillar ones leads to the enhanced fluorescence emission. We observed that the increasing concentration of NaCl can enhance both one- and two-photon excited autofluorescence of HEWL amyloids due to the triggered changes in the morphology and the secondary structure. The recorded ATR-FTIR spectra showed clearly that with the increase of salt concentration the content of secondary β-sheet structure increases. These dependences emphasize the role of H-bonding network which stabilizes the β-sheet structure, enables the possible charge and proton transfer, and stabilizes the nπ* states. With the increase of ionic strength the amyloid fibrils exhibit more efficient autofluorescence excitation and emission intensities. Thus, autofluorescence intensity can differentiate not only between monomers and fibrils but also between different morphologies of amyloids.

Surprisingly, we find that radiative relaxation pathways of one- and two-photon excited fluorescence of amyloids are different, although both 1P and 2P absorption appear to result from the transition to states of the same energy. 2PEF emission spectra are red-shifted comparing to 1PEF ones and 2PEF fluorescence lifetimes are much shorter than those for one-photon excitation. The same behavior was observed in insulin fibrils, suggesting it as a general property of two-photon autofluorescence in amyloids. Finally, considering various correlations between the salt-induced secondary protein structures and optical properties in HEWL monomers and fibrils, we indicate the great potential of these optical processes in sensitive recognition between monomers and fibrils as well as of various amyloid fibrils structures.

## Experimental Methods

*Preparation of the Samples*. Hen egg-white lysozyme (HEWL) was purchased from Sigma-Aldrich (L6876). HEWL was dissolved in HCl solution (pH ∼ 1.5) with varying salt content, namely 0, 5, and 50 mM NaCl. The final concentration of HEWL was adjusted to be 20 mg/mL (1.4 mM). The samples were incubated in an Eppendorf Mixer C for 18 h at 85 °C, following agitation set to 1400 rpm.

*Atomic Force Microscopy (AFM)*. For AFM imaging the samples were diluted to 0.01 mg/mL. The droplets of the samples were deposited on a mica layer and after 5 min rinsed with Milli-Q water and dried afterward. The measurements were conducted by using a Veeco Dimension V atomic force microscope in the tapping mode with the SS probe mounted. The average height and width values of fibrils were estimated according to 50 profiles from different amyloid fibrils by using Nanoscope Software 7.30. For each condition at least three images (3.3 μm × 3.3 μm) were analyzed. The mean height value was estimated based on the highest value of measured profiles, whereas the mean width by using the full width at half-maximum (FWHM) method.

*tTest*. The two-sample *t* test was calculated by using OriginPro 2016 software. The following assumptions were chosen: equal variance not assumed, the significance level was set to 0.05.

*Sedimentation Velocity Analytical Ultracentrifugation (SV AUC)*. Sedimentation velocity analytical ultracentrifugation (SV AUC) experiments were performed at 20 °C at 50000 rpm by using a Beckman Coulter ProteomeLab XL-I analytical ultracentrifuge (Beckman Coulter Inc.) equipped with an AN-60Ti rotor and cells with 12 mm path-length charcoal-filled two-channel Epon center pieces. The experiment was performed for three protein concentrations (0.2, 2, and 20 mg/mL) with varying salt content. The absorbance scans were collected at 280 nm (for protein concentration 0.20 mg/mL), 300 nm (2 mg/mL), and 307 nm (20 mg/mL), time-corrected,^48^ and analyzed in SEDFIT 16p36 (https://sedfitsedphat.nibib.nih.gov/) by using a continuous size distribution *c*(*s*) model with a confidence level set on 0.68.^49,50^ The partial specific volume and the theoretical molecular weight of lysozyme as well as the solution density and dynamic viscosity were calculated by using SEDNTERP 3.0.3 (http://www.jphilo.mailway.com/).^51^ The hydrodynamic dimensions were calculated by SEDFIT 16p36. The plots were obtained by using GUSSI 1.4.2 software.^52^

*Attenuated Total Reflectance Fourier-Transform Infrared (ATR-FTIR) Measurements*. ATR-FTIR spectra were measured by using a Vertex 60v spectrometer. The samples were studied at a concentration of 20 mg/mL. The ATR signals from samples were collected 64 times after a background measurement and averaged. The spectra were recorded in the range of 4000–400 cm^–1^ with the resolution equal to 4 cm^–1^.

*Absorption and Fluorescence Spectroscopies*. One-photon absorption spectra were measured with a Jasco V-670 spectrophotometer in quartz cuvettes within the range 320–700 nm. Concentration of HEWL samples was adjusted to be 20 mg/mL. One-photon excited emission (λ_exc_ = 370 nm) and excitation (λ_em_ = 460 nm) spectra of the same samples were acquired on a FluoroMax-4 spectrofluorometer (Horiba Jobin Yvon).

Two-photon excited fluorescence (2PEF) spectra of native proteins and their fibrillar aggregates were recorded with a two-photon microscope setup described before, equipped with a Chameleon Ti:sapphire laser (Coherent Inc.) with ∼100 fs pulses and the repetition rate equal to 80 MHz.^53^ The samples and the references were illuminated through a microscope objective (Nikon Plan Fluor, 40×, NA 0.75), and 2PEF signals were collected in the epifluorescence mode. 2PEF spectra were measured with a Shamrock 303i spectrometer (Andor) equipped with an iDus camera (Andor).

*Power Dependence of Fluorescence Intensity*. To confirm that observed autofluorescence excited by femtosecond laser pulses was of two-photon origin, we performed the measurement of intensity vs excitation power dependence and determined the power exponent *n* (see eq 1). In the equation, *I* stands for two-photon excited autofluorescence intensity and *P* stands for the incident laser average power.
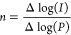
1

*Two-Photon Brightness and Two-Photon Absorption Cross Section*. In our experiment we determined the effective two-photon absorption cross section (two-photon brightness) σ_2,eff_, defined as the absolute 2PA cross section σ_2_ of a fluorophore (a molecule or a particle) multiplied by its fluorescence quantum yield (see eq 2). The effective 2PA cross sections were calculated by using a relative fluorescence technique as described by Makarov et al., where 2PEF of the sample is compared with that of a reference dye with well-known two-photon absorption cross-section values, at the same experimental conditions.^44^
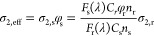
2where *C* is the fluorophore molar concentration per cubic centimeter, *n* the refractive index of the solvent, φ the fluorescence quantum yield, and *F* the integral over the whole two-photon excited emission band. The letters s and r correspond to a sample and a reference, respectively.

*Fluorescence Quantum Yield (FQY)*. The FQY of HEWL monomers and amyloids was measured by using the SC-30 Integrating Sphere Module for a FS5 spectrofluorometer from Edinburgh Instruments. The emission and scattering spectra used in calculations of FQY were measured for excitation set to 360 nm. The concentration of the samples was 20 mg/mL for both monomers and amyloids.

*Fluorescence Lifetime Measurements and Calculations*. One- and two-photon excited fluorescence decays were acquired by time-correlated single-photon counting (TCSPC), the setup containing an Acton SpectraPro SP-2300 monochromator (Princeton Instruments) and a high-speed hybrid detector HPM-100-50 (Becker & Hickl GmbH) controlled by a DCC-100 card. As an excitation source a BDL-375- SMN picosecond laser diode (20 MHz, λ_exc_ = 377 nm) was used. 1PEF decays were measured at λ_em_ = 450 nm. To collect 2PEF decays, the laser diode was replaced with the Chameleon Ti:sapphire femtosecond laser, with 20 MHz repetition rate achieved by using a pulse picker (APE pulseSelect). 2PEF decays were excited at 770 nm and measured at λ_em_ = 470 and 460 nm for HEWL fibrils incubated at 0 and 50 mM NaCl, respectively. Calculated fluorescent lifetimes are an average value from three decays measured for each set of excitation and emission wavelengths. Collected one- and two-photon fluorescence decays were fitted with triple- and double-exponential decay functions, respectively, by using the built-in Nonlinear Curve Fit tool from OriginPro software. Average fluorescence lifetimes (τ_av_) for every decay were calculated based on the fitting results using eq 3:
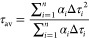
3where α_*i*_ are weights of the contributions of each of the fitted fluorescent lifetimes (τ_*i*_).

*Correction of Inner Filter Effect*. Because of the relatively high concentration of studied samples, we have taken into account the inner filter effect. Therefore, we have calculated corrected fluorescence (*F*_cor_) intensities according to eq 4:^54,55^
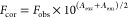
4where *F*_cor_ is the corrected fluorescence intensity, *F*_obs_ is the observed fluorescence intensity, and *A*_exc_ and *A*_em_ are absorbance values at excitation and emission wavelengths.

Then, we calculated the fluorescence enhancement coefficient (*F*_enh,coeff_) values as follows:
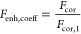
5where *F*_cor,1_ is corrected fluorescence value obtained for monomers or fibrils at 0 mM NaCl, depending on species taken into account. The calculations were performed for 1PEF and 2PEF processes for HEWL monomers and fibrils.

## Supporting Information Available

Detailed information regarding AFM mean height and width distribution among HEWL fibrils obtained after incubation at 0, 5, and 50 mM NaCl; *t* test statistics calculated for height among the possible groups of HEWL fibrils incubated at 0 and 5 mM NaCl, 0 and 50 mM NaCl, and 5 and 50 mM NaCl; height AFM images of HEWL monomers; parameters derived from the SV AUC experiments; SV AUC analysis for HEWL samples revealing predomination of monomers before the incubation period; ATR-FTIR spectra recorded for HEWL monomers at varying NaCl content; absorption spectra recorded for HEWL monomers and fibrils; fluorescence emission spectra recorded for HEWL fibrils at 2 and 20 mg/mL; peak positions of one- and two-photon emission and excitation spectra; two-photon excited fluorescence of HEWL fibrils at 20 and 2 mg/mL excited at 750 nm; one- and two-photon excited lifetimes obtained after fitting the fluorescence decays along with their share in the calculated average lifetime; normalized one- and two-photon excited fluorescence spectra of bovine insulin amyloids; fluorescence emission spectra recorded for HEWL monomers dissolved in 50 mM NaCl at concentration 2 and 20 mg/mL (PDF)
