# Supplementary material for: One- and Two-Photon Excited Autofluorescence of Lysozyme Amyloids
Source: J Phys Chem Lett. 2022 May 23;13(21):4673–81. doi: 10.1021/acs.jpclett.2c00570 (PMC9169060; doi:10.1021/acs.jpclett.2c00570)
Supplement: Supplementary file 1 — jz2c00570_si_001.pdf [file jz2c00570_si_001.pdf]

Supporting Information

One- and Two-Photon Excited Autofluorescence of Lysozyme Amyloids

*Manuela Grelich-Mucha [a], Maciej Lipok [a], Mirosława Różycka [b], Marek Samoć [a], Joanna Olesiak-Bańska [a]**

[a] Advanced Materials Engineering and Modelling Group, Wroclaw University of Science and Technology, Wybrzeże Wyspiańskiego 27, 50-370 Wrocław, Poland.

[b] Department of Biochemistry, Molecular Biology and Biotechnology, Faculty of Chemistry, Wroclaw University of Science and Technology, Wybrzeże Wyspiańskiego 27, 50-370 Wrocław, Poland.

Corresponding Author

E-mail: joanna.olesiak@pwr.edu.pl

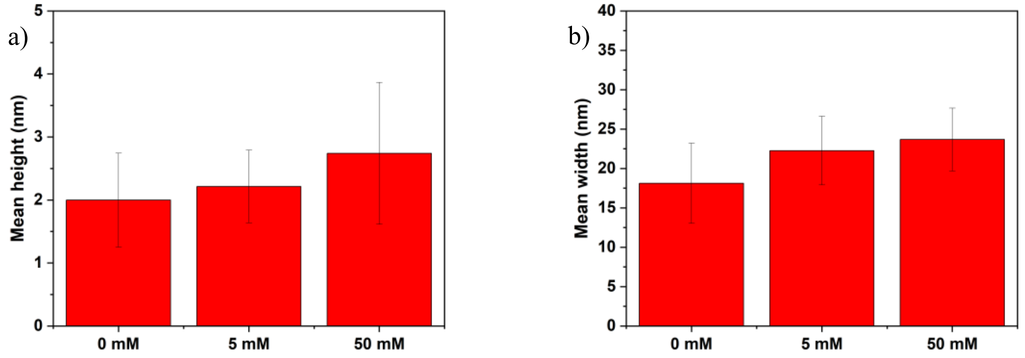

Figure S1. Mean height (a) and width (b) distribution among amyloid fibrils obtained after incubation of HEWL at 0 mM, 5 mM, and 50 mM NaCl. The average height and width values were calculated according to 50 profiles from different amyloid fibrils.

Table S1. T-test statistics calculated for height among the possible groups of HEWL fibrils incubated at 0 mM and 5 mM NaCl, 0 mM and 50 mM NaCl, 5 mM and 50 mM NaCl.

Group of samples	t Statistic	Prob>|t|
**0 mM NaCl**<br>**5 mM NaCl**	-1.62048	0.10854
**0 mM NaCl**<br>**50 mM NaCl**	-3.88849	1.99442E-4
**5 mM NaCl**<br>**50 mM NaCl**	-2.94118	0.00438

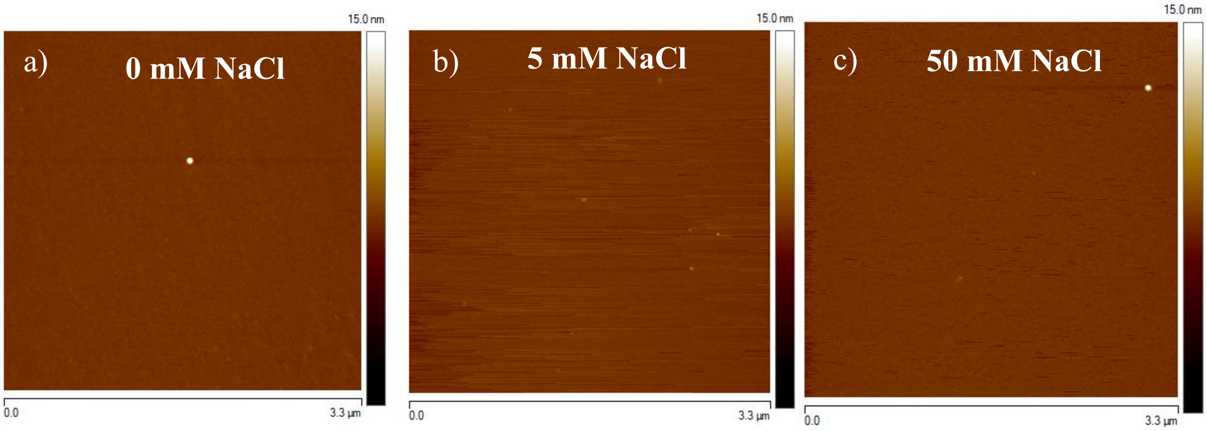

Figure S2. Height AFM images performed for HEWL samples before incubation at varying salt concentration: 0 mM (a), 5 mM(b), and 50 mM NaCl (c). The size of the images is 3.3 µm x 3.3 µm and the height contrast is set to 15 nm.

Table S2. Parameters derived from the SV AUC experiments. Abbreviations: s, sedimentation coefficient; $s_{20,w}$, sedimentation coefficient corrected for water at 20°C; f/f0, frictional ratio; R_S, Stokes radius; MW_{app}, apparent molecular weight; MW_{theor}, theoretical molecular weight; RMSD, root mean square deviation.

**c (mg/mL)**	**NaCl (mM)**	***s* (S)**	**$s_{20,w}$ (S)**	**f/f_0**	**R_S (nm)**	**MW_{app} (kDa)**	**% of signal**	**RMSD**
**0.2**	0	1.826	1.828	1.241	1.98	14.39	99.79	0.00690
	5	1.816	1.823	1.251	2.00	14.50	99.85	0.00746
	50	1.782	1.803	1.252	1.99	14.28	99.92	0.00691
**2.0**	0	1.739	1.741	1.306	2.09	14.45	99.63	0.00879
	5	1.750	1.757	1.301	2.09	14.56	99.52	0.00764
	50	1.721	1.743	1.313	2.11	14.59	99.50	0.00857
**20.0**	0	1.209	1.214	1.859	2.96	14.28	95.50	0.01279
		4.320	4.336		5.60	96.44	3.463	
	5	1.209	1.213	1.869	2.99	14.39	96.46	0.01322
		4.435	4.452		5.72	101.13	2.383	
	50	1.395	1.412	1.600	2.55	14.31	98.91	0.01199

**MW_{theor} (kDa)**
14.30

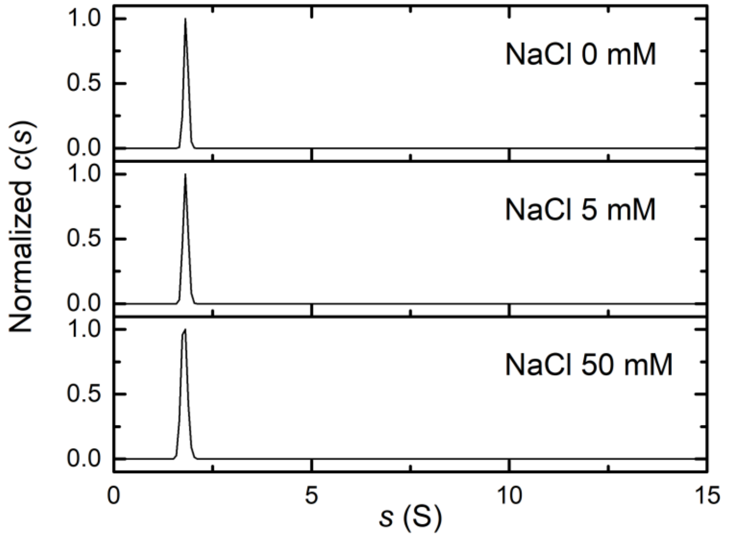

Figure S3. SV AUC analysis performed for HEWL samples before the incubation period revealing predomination of monomers. Normalized sedimentation coefficient distributions *c*(*s*) of HEWL in the concentration of 0.2 mg/mL without and in the presence of 5 mM and 50 mM NaCl.

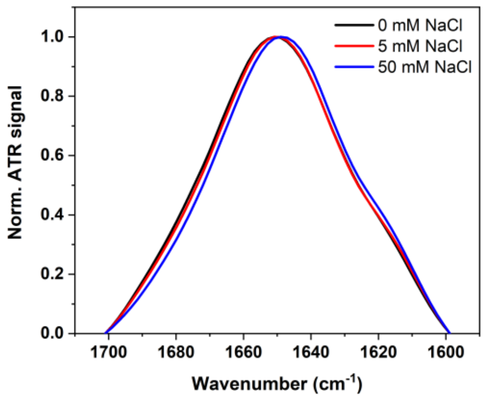

Figure S4. ATR-FTIR spectra recorded for HEWL samples before the incubation period at varying salt concentration: 0 mM (black), 5 mM (red), 50 mM (blue).

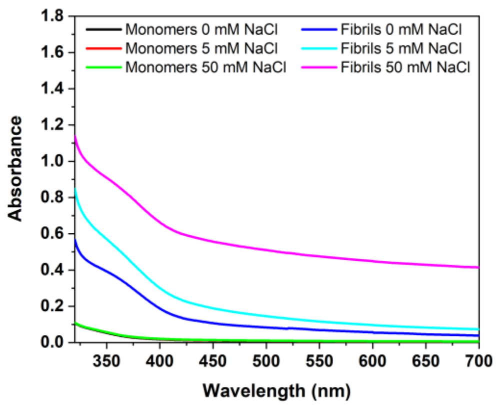

Figure S5. Absorption spectra recorded for monomers at 0 mM (black), 5 mM (red), and 50 mM (green) NaCl, for fibrils at 0 mM (blue), 5 mM (cyan) and 50 mM NaCl (magenta). The absorption spectra were recorded in the range 320-700 nm.

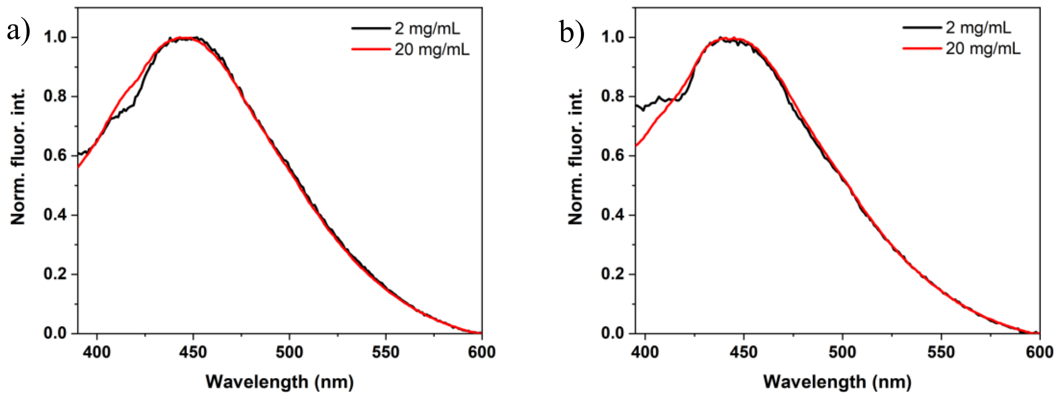

Figure S6. Fluorescence emission spectra recorded for HEWL fibrils incubated at 5 mM (a) and 50 mM NaCl (b). Emission spectra were recorded at λ_{exc}=370 nm and measured for the samples at 2 mg/mL (black) and 20 mg/mL (red).

Table S3. Peak positions of one- (1P) and two-photon (2P) emission (1PEF and 2PEF) and excitation spectra (1P exc and 2P exc). The spectra were measured for HEWL fibrils incubated in the absence of salt.

Process	Peak position (nm)
1PEF	451
2PEF	488
1P exc	373
2P exc	750

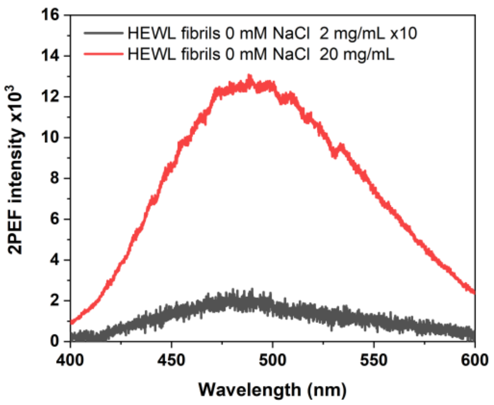

Figure S7. Two-photon excited fluorescence of HEWL fibrils incubated at 0 mM NaCl with two concentrations of protein: 20 mg/mL (red) and 2 mg/mL (grey) excited at 750 nm.

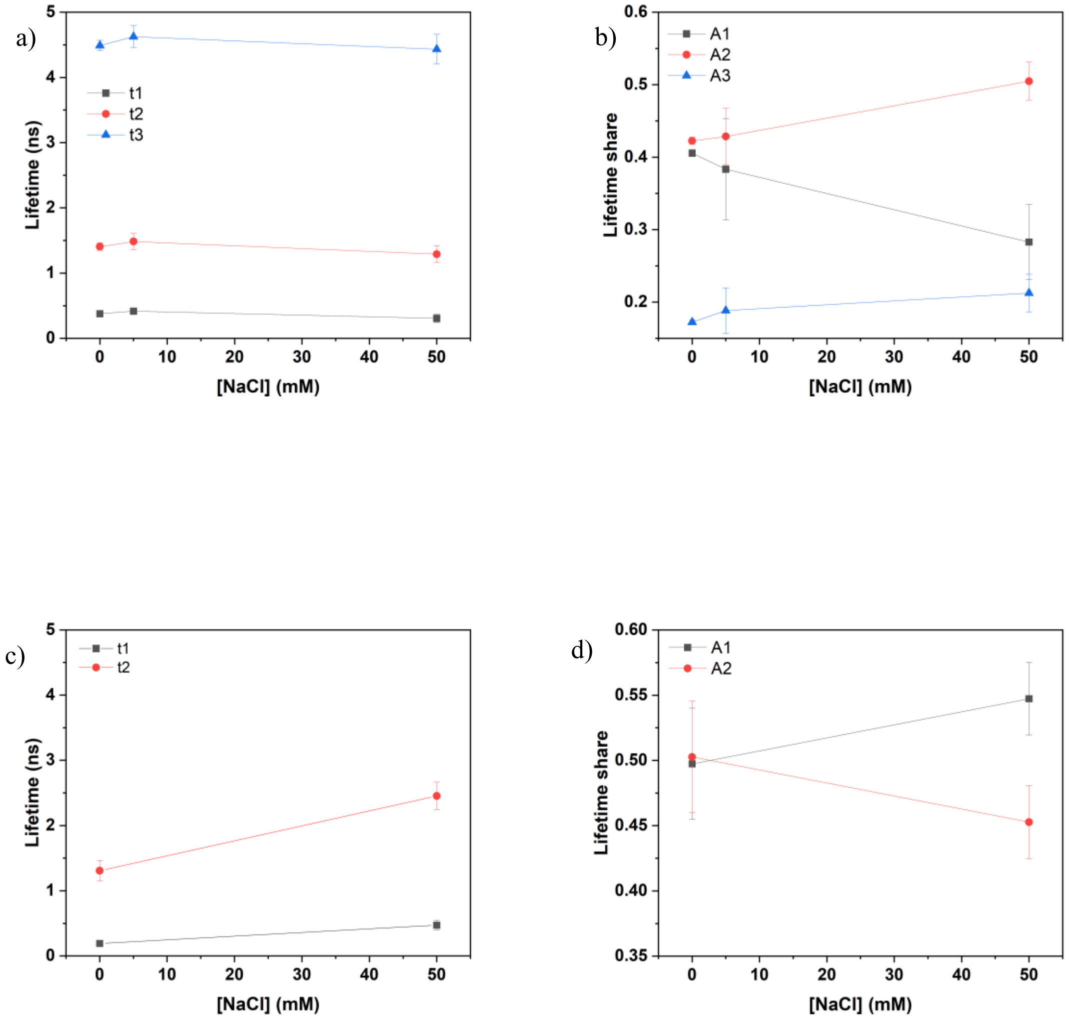

Figure S8. One (a) and two-photon excited lifetimes (c) obtained after fitting the fluorescence decays along with their share (b and d, respectively) in the calculated average lifetime in respect to the salt concentration.

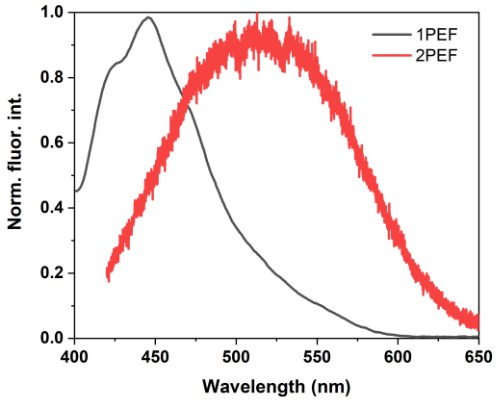

Figure S9. Normalized one-photon and two-photon excited fluorescence spectra of bovine insulin amyloids. One-photon excited spectra were collected using excitation wavelength at 375 nm and two-photon excited spectra were collected using 750 nm.

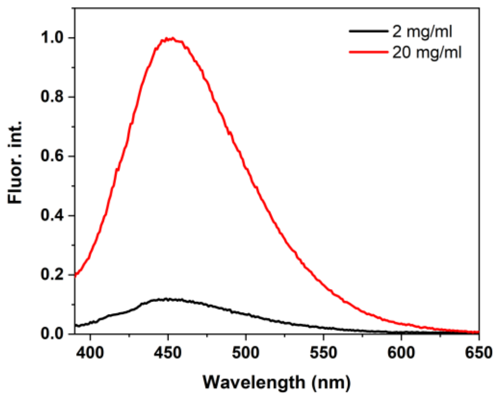

Figure S10. Fluorescence emission spectra (λ_{exc}=370 nm) recorded for HEWL monomers dissolved in 50 mM NaCl at concentration 2 mg/mL (black) and 20 mg/mL (red). The spectra were obtained by dividing by the maximum emission intensity recorded for the sample at 20 mg/mL.
